# The association between gestational diabetes and fear of childbirth: a longitudinal register study

**DOI:** 10.1186/s12884-024-07022-9

**Published:** 2024-12-18

**Authors:** Josephine Savard, Guro Pauck Bernhardsen, Anu Mykkänen, Leea Keski-Nisula, Soili Marianne Lehto

**Affiliations:** 1 Research and Development Department, Division of Mental Health Services, Akershus University Hospital, Lørenskog, Norway; 2 Department of Medicine, Karolinska Institutet, Stockholm, Sweden; 3 Institute of Clinical Medicine, School of Medicine, University of Eastern Finland, Kuopio, Finland; 4 Department of Obstetrics and Gynecology, Kuopio University Hospital, Kuopio, Finland; 5 Institute of Clinical Medicine, University of Oslo, Oslo, Norway; 6 Department of Psychiatry, University of Helsinki and Helsinki University Hospital, Helsinki, Finland

**Keywords:** Pregnancy, Diabetes, Gestational, Fear of childbirth, Depression, Prenatal care

## Abstract

**Background:**

Gestational diabetes mellitus is a common condition known to be associated with pregnancy complications, larger fetus size and depression, and may therefore lead to increased concerns linked to childbirth. We sought to determine whether gestational diabetes mellitus is linked to fear of childbirth, and whether the possible association is mediated by depressive symptoms.

**Methods:**

This study includes women who gave birth at the Kuopio University Hospital between 2019–2022 and had reported their level of fear of childbirth after gestational week 28 (*n* = 3293). Two outcome measures of fear of childbirth were used: self-rated intensity on a visual analogue scale, and obstetrician-confirmed diagnosis. Gestational diabetes mellitus was diagnosed based on plasma glucose concentrations in fasting state (≥ 5.3 mmol/l) and after a 75 g glucose load (one hour: ≥ 10.0 mmol/l, two hours: ≥ 8.6 mmol/l). The Edinburgh Postnatal Depression Scale (EPDS) was used to assess depressive symptoms in the third trimester. We performed logistic and linear regression analyses while adjusting for possible confounding factors and examined the controlled direct effect by including depressive symptoms in the model.

**Results:**

Gestational diabetes mellitus was associated with increased risk of fear of childbirth diagnosis (OR = 1.42, 95% CI 1.11─1.73) and higher levels of fear of childbirth (B = 0.31, 95% CI 0.09─0.53), but the associations were attenuated and no longer significant after further adjustments for body mass index and health behaviors (OR = 1.22, 95% CI 0.91─1.5; B = 0.11, 95% CI -0.13─0.35). Inclusion of depressive symptoms in the model attenuated the non-significant estimates further.

**Conclusions:**

The observed association between fear of childbirth and gestational diabetes mellitus in previous studies may result from the lack of adjustments for confounding factors.

**Supplementary Information:**

The online version contains supplementary material available at 10.1186/s12884-024-07022-9.

## Introduction

In parallel to the global obesity epidemic [1], the prevalence of gestational diabetes mellitus (GDM), currently up to 8.2% in the United States, appears to be on the rise [2]. GDM is associated with various pregnancy and perinatal complications such as pre-eclampsia, macrosomia, and increased need for Caesarean Section [3]. The management of GDM includes sharing information about the risks and the importance of health behaviors and glycemic control [3]. This information, while imperative for the women’s wellbeing, may also lead to heightened psychological stress and increased fear of childbirth (FOC).

There is no universally accepted definition for FOC. In broad terms, FOC can be described as various emotional difficulties linked to giving birth [4]. Its global pooled prevalence has been estimated to be 14% [5], although the prevalence varies across countries, possibly due to different measures and definitions of FOC [4]. Nonetheless, FOC has been associated with consequences such as prolonged labor [6], and post-partum depression [7].

In general, most studies that have investigated factors predisposing to FOC have focused on sociodemographic and psychological factors, and aspects related to previous births, while less attention has been paid to maternal physical health [8–13]. To the extent of our knowledge, only a few studies have addressed GDM as a potential risk factor for FOC with reports of an increased prevalence of GDM among women with a physician-diagnosed FOC [11, 14]. However, these papers applied limited to no adjustments for potential confounding factors. GDM is profoundly influenced by body mass index (BMI) and health behaviors such as low levels of physical activity [15, 16], which could either directly or indirectly influence FOC.

Furthermore, previous studies have suggested that depression is a strong risk factor for FOC [8, 11, 17], and bi-directionally associated with GDM [18]. Therefore, depression may mediate the association between GDM and FOC; concerns related to the GDM diagnosis itself, and fear of perinatal and infant complications may all lead to depressive symptoms and a subsequent FOC [18, 19].

In conclusion, both GDM and FOC conditions have overlapping risk factors, are associated with adverse health outcomes, and have an impact on labor and the postpartum period. A better understanding on factors influencing these conditions will provide guidance to improve the well-being of pregnant women. Subsequently, the specific aim of this study was to investigate whether GDM is linked to *diagnosis and self-reported intensity of FOC* while adjusting for possible confounding factors. A secondary aim was to examine whether a possible association between GDM and FOC may be mediated by depressive symptoms.

## Methods

### Data collection and study sample

**Fig. 1:**
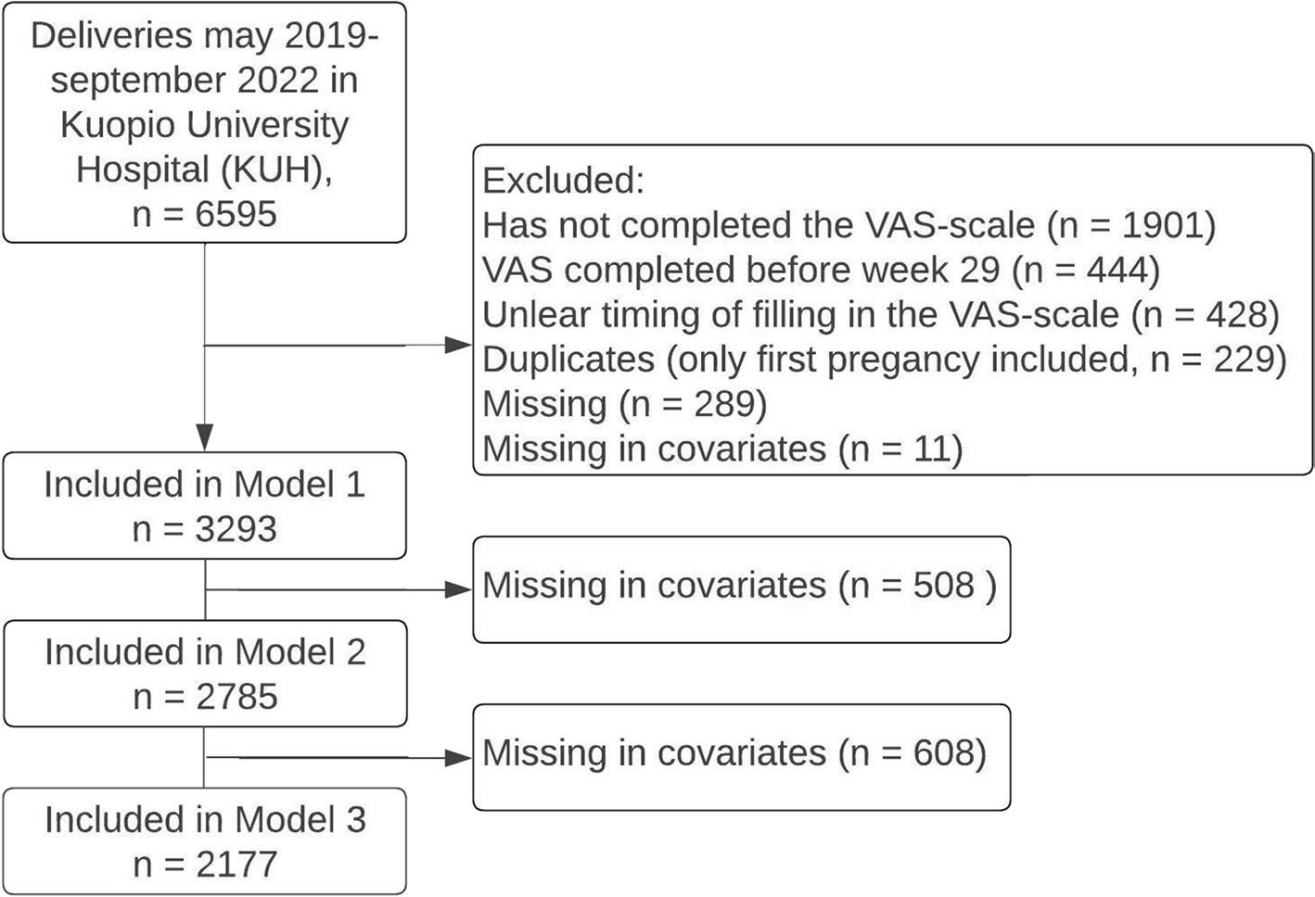
Flowchart

This study is based on births between May 2019 and September 2022 at the Kuopio University Hospital (KUH), a tertiary care university hospital that, together with three secondary care hospitals, is responsible for labors for a population of one million in the region of Central-Eastern Finland. As a tertiary care hospital, The KUH is responsible for risk pregnancies in the area. Information regarding the births was derived from the KUH Birth Register and covered demographic and clinical data of participating women and their pregnancies (that is, age, weight, height, relationship status, gravidity, parity, gestational age, smoking, and diagnoses). Data in the registry is recorded by the health care professionals as well as by the women themselves via questionnaires. The following pregnancies were included in the study cohort: 1) Pregnancies where all questionnaires included in the primary analyses (Model 1) were completed before delivery; 2) Pregnancies where the mother had self-reported the intensity of FOC on a visual analogue scale (VAS) after gestational week 28 (that is, when GDM most likely have been diagnosed); 3) The first pregnancy of each woman included in the database. Please see Fig. 1 for a more detailed description of the selection of study participants.

### Measurements

#### Gestational diabetes mellitus

In Finland and internationally, clinical guidelines recommend screening for GDM during pregnancy weeks 24–28 in women not previously diagnosed with diabetes [20, 21]. In Finland, in women with a high risk of GDM (that is, BMI ≥ 30 kg/m^2^, waist circumference > 90 cm, previously diagnosed non-alcoholic fatty liver disease, previous gestational diabetes, glucosuria at the beginning of pregnancy, presence of type 2 diabetes in parents, siblings or children, or oral corticosteroid medication), an oral glucose tolerance test (OGTT) is performed between weeks 12–16, or in some cases earlier. There are two exceptions for performing OGTT: 1) nulliparous women under 25 years with BMI < 25 kg/m^2^ and without close relatives (that is, siblings, parents, or grandparents) with type 2 diabetes mellitus and 2) multiparous women under 40 years with BMI < 25 kg/m^2^ and without a history of GDM and fetal macrosomia. Furthermore, if the pregnant woman develops features associated with GDM (for example, excessive increase in weight gain, amniotic fluid volume or fetal growth), an OGTT is performed. This also includes individuals with previously normal OGTT values. Procedures for the OGTT are as follows: after a 12-h fast, the women drink 300 ml of water containing 75 g of glucose within five minutes. Venous blood samples for glucose levels are collected before, and one and two hours after the ingestion. Diagnostic thresholds are 5.3 mmol/l for the fasting stage, 10.0 mmol/l for one hour, and 8.6 mmol/l for two hours after drinking the solution. A glucose level equal to or above the set value is deemed diagnostic for GDM.

#### Fear of childbirth

Women were asked about FOC by their primary health care provider in maternity outpatient clinics during the first trimester, and, when needed, referred to specialized FOC clinics.

FOC was diagnosed according to the International Classification of Diseases 10th revision (ICD-10) O99.80. other specified diseases and conditions complicating pregnancy, childbirth, and the puerperium, established in 1997 in Finland [22]. The diagnosis is given to individuals with a moderate-to-severe FOC that affects daily functioning and sleep. Physicians evaluate the patients for FOC when they are referred to specialized FOC perinatal clinics, or if the fear is identified and addressed during a maternity care visit. In Finland, O99.80 is solely used for FOC. The diagnoses were recorded and extracted from birth registry. Women whose symptom profiles were dominated by depression or anxiety not specific to childbirth were referred for psychiatric evaluation.

For all participants, the level of FOC was also assessed on a VAS after gestational week 28. The women were asked to “Rate the intensity of your fear of childbirth using the scale below (0 = no fear, 10 = uncontrollable fear)”. Previously, VAS ≥ 5 has demonstrated a sensitivity of 97.8% and specificity 65.7% to identify women with severe FOC on the reference test Wijma Delivery Expectancy/Experience Questionnaire (W-DEQ ≥ 100) [23].

#### Depression

Depressive symptoms were assessed using the 10-item Edinburgh Postnatal Depression Scale (EPDS). Each item (i.e. symptom) is scored 0–3 and the total score range is 0─30, with ≥ 10 indicating elevated symptoms of depression [24]. The EPDS has no definitive accepted cut-off value, and sensitivity and specificity estimates have varied in different studies due to considerable differences in the methodology [25]. However, a cut-off of 9/10 has been suggested in clinical or research settings, given a failed detection rate of less than 10% [24]. The women completed EPDS during the first and third trimester; the results from third trimester were used in the mediation analyses, whereas first trimester was only used in descriptive statistics. Information on diagnoses of depression was retrieved from ICD-10 codes derived from the birth registry [22].

#### Covariates

Women were classified as nulliparous (that is, as having no previous childbirths) vs. primi or multiparous (≥ 1 previous childbirth). The women self-reported background characteristics, through an online questionnaire during the first trimester. Self-reported living situation (that is, living with a partner, alone or other) was used as a proxy measure of socioeconomic status, whereas alcohol use and smoking were considered as measures of health behaviors. Smoking habits during pregnancy were self-reported during the third trimester, and categorized as nonsmoking; quit smoking during pregnancy, and smoking. Alcohol use was assessed in the first trimester with The Alcohol Use Disorders Identification Test (AUDIT) [26], a 10-item scale with questions on drinking behavior, alcohol use, and alcohol-related problems during the last 12 months. The score range: 0–40, with ≥ 6 indicating harmful use in women. In addition, the women answered questions regarding the quantity of alcohol consumed before and during pregnancy in standard alcohol units (e.g., 1 unit = 12 cl wine or 4 cl spirits).

A subsample of participants self-reported antidepressants use covering pregnancies from 2019 to 2021. Among the participants in the subsample (*n* = 2318), 5.6% reported antidepressant use.

### Statistical analyses

Descriptive statistics of participants stratified by women with and without FOC diagnosis during pregnancy are presented as means and standard deviations (SDs) or medians and interquartile ranges (IQR) based on the distribution of the data. Differences between these two groups were analyzed using chi-square tests, Fisher’s exact test, and Mann–Whitney U tests.

To examine the associations between GDM and the two outcome measures; the diagnosis of FOC and the intensity of FOC, we used multiple logistic regression and multiple linear regression, respectively. We tested the multiple linear regression analyses for normal distribution of residuals and homoscedasticity and tested all included variables for multi-collinearity.

We drew a directed acyclic graph (DAG), a conceptual illustration of the relationships between factors relevant to the current study, to identify possible confounding factors, that is, factors that might influence the development of both GDM and FOC, according to the previous literature (Supporting Information, Figure S1) [27]. There is unconclusive evidence whether antidepressants use can increase the risk of GDM, and whether it therefore represents a confounding factor for the association between GDM and FOC. An observed increased risk of GDM may represent confounding by indication [28], or differential impact of distinct antidepressants. Furthermore, a Norwegian nation-wide cohort study found no association between the most used antidepressants (Selective Serotonin Reuptake Inhibitors and Serotonin and Norepinephrine Reuptake Inhibitors, which have low histamine-1(H_1_) receptor affinity) and GDM [29]. Antidepressants with high H_1_receptor affinity may, however, raise GDM risk; nevertheless, adjusted analyses were limited due to low statistical power [29]. In our subsample, only 0.47% (11/2318) reported using high H_1_ affinity antidepressants. Therefore, we did not anticipate that antidepressants would influence the results, and therefore did not include them in the main analyses.

To examine the impact of subclasses of confounding factors, as well as the impact of depressive symptoms as a possible mediating factor, we performed the regression analyses in three steps: In Model 1 we adjusted for age, parity and living situation. To further investigate whether health behaviors and BMI confounded the investigated associations, we additionally included alcohol, smoking and BMI in Model 2. In Model 3, we further evaluated whether an association may be mediated by depressive symptoms in the 3rd trimester (that is, we examined the controlled direct effect) [30]. Some additional confounding and interaction assumptions are required when examining the controlled direct effect [30], including mediator-outcome confounders and interaction between exposure and the mediator. Antidepressants may represent a mediator-outcome confounder by impacting both depressive symptoms and FOC. We therefore ran sensitivity analyses including antidepressant use (yes/no) in Model 3 and tested for interaction between GDM and depressive symptoms. Additionally, to evaluate the individual impact of BMI on the previous associations, we also performed sensitivity analyses with the variables in Model 1 and only BMI from Model 2.

A *P*-value < 0.05 was considered statistically significant. The data were analyzed using SPSS (version 29) and Stata/SE 17.0.

## Results

### Study sample

The cohort consisted of 3293 deliveries (52% of births in the registry; baseline characteristics of excluded women is presented in Supporting Information, Table S1). Among women in our cohort, 13% (*n* = 442) had a diagnosis of FOC and 26% (*n* = 849) had GDM.

Women with FOC had higher prevalence of GDM (cases 32%, reference group 25%), higher BMI, and their babies had a lower gestational age at delivery compared with the women without FOC (Table 1). Previous GDM was more common in primi-multiparous women with FOC compared with primi-multiparous without FOC (cases 29%, reference group 23%, *P* = 0.042). Women with FOC reported more depressive symptoms and more often had a diagnosis of depression compared with women without FOC. In addition, women with FOC reported lower alcohol consumption before pregnancy, but there were no differences in the AUDIT scores or in alcohol use during pregnancy (Table 1).

**Table 1:** Demographic and clinical characteristics of women with diagnosis of fear of childbirth (*n* = 442) and reference group (*n* = 2851). All values are given as n (%) unless indicated otherwise

Characteristics	FOC	Reference group	Test Statistics	*P*-value
Maternal age, *mean (SD)*	31.1 (5.6)	30.8 (5.3)	Z = 0.81	0.419
Living situation				
Living with a partner	406 (92)	2672 (94)	χ^2^ = 4.25	0.120
Alone	13 (3)	86 (3)		
Other	23 (5)	93 (3)		
Parity				
Nulliparous	190 (43)	1332 (47)		
Primi-multiparous	252 (57)	1519 (53)	χ^2^ = 2.15	0.143
Number of fetuses				
> 1	6 (1)	52 (2)	χ^2^ = 0.48	0.488
Gestational age at delivery (weeks), *mean (SD)*	38.9 (1.5)	39.2 (1.6)	Z = −4.87	** < 0.001**
*Health variables*				
BMI 1st trimester (kg/m^2^), *mean (SD)*^a^	26.7 (6.1)	25.9 (5.5)	Z = 2.70	**0.007**
Alcohol use before pregnancy (units/week), *median (IQR)*	0 (1)	0 (1)	Z = −2.13	**0.033**
Alcohol use during pregnancy ^b^				
Never	390 (95)	2650 (97)		
Once a month or more often	19 (5)	82 (3)	χ^2^ = 3.09	0.079
Smoking status ^c^				
Non-smoking	336 (81)	2076 (83)		
Quit smoking during pregnancy	57 (14)	342 (14)		
Smoking	20 (5)	75 (3)	χ^2^ = 3.81	0.149
*Comorbid medical conditions*				
Diabetes mellitus	5 (1)	31 (1)	FET	0.809
Gestational diabetes	142 (32)	707 (25)	χ^2^ = 10.74	** < 0.001**
Previous gestational diabetes (primi-and multiparous only)	72 (29)	345 (23)	χ^2^ = 4.12	**0.042**
Preeclampsia	16 (4)	93 (3)	χ^2^ = 0.15	0.696
Depression diagnosis	17 (4)	45 (2)	χ^2^ = 10.65	**0.001**
Anxiety diagnosis	7 (2)	20 (1)	FET	0.080
*Questionnaires*	*median, (IQR)*	*median, (IQR)*		
FOC score	8 (1)	3 (4)	Z = 28.01	** < 0.001**
EPDS score 1st trimester ^d^	5 (6)	3 (5)	Z = 3.85	** < 0.001**
EPDS score 3rd trimester ^e^	6 (7)	4 (5)	Z = 8.53	** < 0.001**
AUDIT	2 (3)	3 (2)	Z = −1.10	0.271

1 unit alcohol = e.g., 12 cl wine or 4 cl spirits. *Test statistics:* FET = Fisher’s exact test; χ^2^ = Chi-square test; Z = Mann–Whitney U test

*Abbreviations: AUDIT* Alcohol Use Identification Test, *BMI* Body mass index, *FOC* Fear of childbirth, *EPDS* Edinburgh Postnatal Depression Scale, *IQR* Interquartile range, *SD* Standard Deviation

^a^Data available on 3275 women

^b^Data available on 3141 women

^c^Data available on 2906 women

^d^Data available on 1330 women

^e^Data available on 2528 women

^f^Data available on 2487 women

### Fear of childbirth, gestational diabetes, and depression

**Fig. 2:**
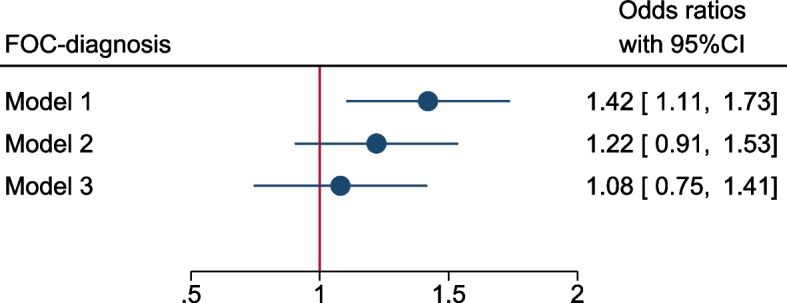
Likelihood of women with gestational diabetes to be diagnosed with fear of childbirth

**Fig. 3:**
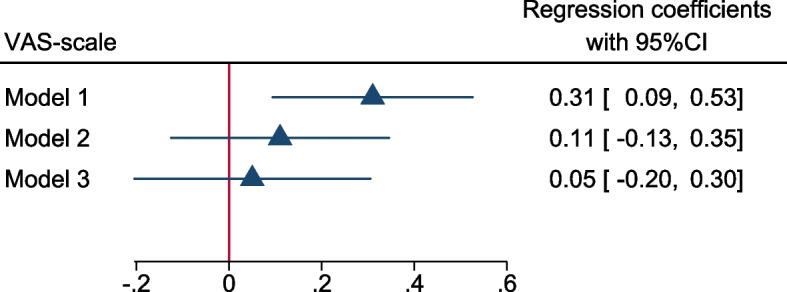
Gestational diabetes diagnosis prediction of the level of fear of childbirth

Figures 2 and 3 illustrate the results of the logistic and linear regression models. In Model 1 GDM was an independent predictor of FOC diagnosis (adjusted odds ratio (aOR) = 1.42, *P* = 0.002), but GDM did not withstand as an independent predictor in Model 2 (aOR = 1.22, *P* = 0.116), or Model 3 (aOR = 1.08, *P* = 0.614, Fig. 2). Sensitivity analyses including antidepressant use in Model 3 had little impact on the results (OR = 1.1, 95%CI = 0.8; 1.5), and there was no interaction between GDM and depressive symptoms (*P* = 0.359).

Similarly, GDM was found to be an independent predictor of the level of FOC, as measured with the VAS scale, in the linear regression Model 1 (B = 0.31, *P* = 0.004), but not in either Model 2 (B = 0.11, *P* = 0.358) or Model 3 (B = 0.05, *P* = 0.715, Fig. 3). The sensitivity analyses showed that additional adjustments for antidepressant use had no impact in Model 3 (B = 0.04, 95%CI = −0.24; 0.32), and there was no interaction between GDM and depressive symptoms (*P* = 0.149).

Sensitivity analyses including BMI and the variables from Model 1 resulted in an OR = 1.32 (95% CI 1.04─1.68, *P* = 0.020) for FOC diagnosis and B = 0.17 (95% CI = −0.06 to 0.40, *P* = 0.151) for the level of FOC.

## Discussion

In accordance with previous studies [11, 14], we found in the initial analyses that women with FOC were more likely to have GDM; Vaajala et al. [14] reported an increased prevalence of GDM among women with a physician-diagnosed FOC, using data from one million pregnancies derived from the Finnish National Medical Birth Register during 2004─2018. Similarly, Räisänen et al. [11] also observed an association between GDM and FOC, utilizing data derived from the same register during a partially overlapping period (1997–2010).

However, the previous studies reporting an association between GDM and FOC did not specifically aim to investigate this association, and hence did not control for BMI and health behaviors, which could explain the discrepancy in the results. More specifically, our sensitivity analyses suggested that the associations between GDM and the level of FOC were largely explained by BMI as a possible confounding factor. BMI is a main risk factor for GDM [15], however; the link with FOC is less consistent [4]; some previous studies support an association between bodyweight and FOC [14, 31], whereas others do not [8, 32]. Nonetheless, the link is remarkably unexplored as it may be assumed that risks associated with overweight during pregnancy (for example, maternal health complications, larger fetus size, unfavorable fetal and neonatal outcomes, and risks at the time of delivery) [33], could lead to worries and subsequently FOC. Therefore, more studies examining the impact of BMI on FOC are warranted.

Moreover, when we included depressive symptoms in the analyses under the hypothesis that depression was a potential mediator, the association coefficients between GDM and FOC approached zero (Model 3). Depression has previously been reported as a risk factor of FOC [8, 11], and bi-directionally associated with GDM [18]. Therefore, depression may also be a potential confounder of the association between GDM and FOC, although to confirm this hypothesis, depression would need to be assessed before a diagnosis of GDM and FOC. In our sample, women with FOC reported higher scores on the EPDS in the first trimester, although the number of missing cases and the descriptive nature of data warrants caution when drawing conclusions.

Strengths of this study include the use of a cohort with information on several covariates, multiple measures of FOC, the thorough laboratory- and physician-confirmed diagnosis of GDM, as well as the use of a structural visual graph (i.e., DAG) to identify confounders and mediators.

This study also has limitations; although we controlled for several factors that are likely to influence the link between GDM and FOC, there are likely to be additional confounding factors that remain unaccounted for. For example, we used living situation as a proxy measure of socioeconomic status, though it would have been desirable to include information about educational level and income. Similarly, health behaviors would have ideally been determined via more detailed measures regarding diet quality and objective, device-measured physical activity. Some variables are based on self-report and may as such be prone to measurement error. Nevertheless, the fact that the self-report data was part of the pregnant women’s usual prenatal care may have increased the accuracy of the reporting. Furthermore, since the exposure was objectively measured with the OGTT, the measurement errors for the exposure and outcome are likely independent and non-differential [34].

Further, although we restricted the inclusion to women who filled in VAS questionnaire after week 28 (that is, after most women have participated in the OGTT and received the diagnosis of GDM), some cases might have received the diagnosis of GDM later. Information concerning FOC in early pregnancy, clearly before a possible diagnosis of GDM, would have allowed for more solid conclusions.

Finally, as our inclusion criteria were determined to address our main hypothesis, it limited the women who could be included in the analyses. The included women did not differ from the excluded women with respect to age or prevalence of gestational diabetes; however, there were a smaller percentage of nulliparous woman (of note, we only included the first pregnancy recorded in the cohort for each woman), and women with a diagnosis of FOC in the excluded cohort. As approximately 9% of pregnant women in 2018 were diagnosed with FOC according to the Finnish National Medical Birth Register [14], there might be an overrepresentation of FOC in the study cohort. It may be that women who did not complete the VAS questionnaire are less prone to experience FOC; this needs to be considered while interpreting our results.

## Conclusion

GDM does not appear to be a main and independent risk factor for FOC. However, more research on the possible impact of BMI and health behaviors on FOC is warranted. A clear understanding of how these factors influence FOC would provide guidance on preventive and supportive interventions to women at risk of developing FOC.

## Supplementary Information

Supplementary Material 1.

## Data Availability

The data that support the findings of this study are available from Kuopio University Hospital but restrictions apply to the availability of these data, which were used under license for the current study, and so are not publicly available. Data are however available from the authors upon reasonable request and with permission of Kuopio University Hospital.

## Abbreviations

AUDIT: The Alcohol Use Disorders Identification Test
BMI: Body mass index
CI: Confidence interval
EPDS: Edinburgh Postnatal Depression Scale
FOC: Fear of childbirth
GDM: Gestational diabetes mellitus
KUH: Kuopio University Hospital
OR: Odds ratio
OGTT: Oral glucose tolerance test
VAS: Visual analogue scale
