# Supplementary material for: The association between gestational diabetes and fear of childbirth: a longitudinal register study
Source: BMC Pregnancy Childbirth. 2024 Dec 18;24:814. doi: 10.1186/s12884-024-07022-9 (PMC11654169; doi:10.1186/s12884-024-07022-9)
Supplement: Supplementary file 1 — Supplementary Material 1. [file 12884_2024_7022_MOESM1_ESM.docx]

## **Supporting Information**

**The association between** **gestational diabetes and fear of childbirth: A longitudinal register study**

Josephine Savard,^1,2 *^ Guro Pauck Bernhardsen,^1^ Anu Mykkänen,^3,4^ Leea Keski-Nisula, ^3,4^Soili Marianne Lehto, ^1,5^

^1^ Department of Research and Development, Division of Mental Health Services, Akershus University Hospital, Lørenskog, Norway

^2^ Department of Medicine, Karolinska Institutet, Stockholm, Sweden

^3^ Institute of Clinical Medicine, School of Medicine, University of Eastern Finland, Kuopio, Finland

^4^ Department of Obstetrics and Gynecology, Kuopio University Hospital, Kuopio, Finland

^5^ Institute of Clinical Medicine, University of Oslo, Oslo, Norway

* Corresponding author: [Josephine.savard@ki.se](mailto:Josephine.savard@ki.se)

**Table S1. Characteristics of included and excluded women.**

|  | | |  |  |
| --- | --- | --- | --- | --- |
|  | Included n = 3293 | Excluded  n = 3013 | Test Statistics | P-value |
| Maternal age, *mean (SD)* | 30.9 (5.4) | 31.0 (5.3) | 1.21 | 0.226 |
| Living situation, (%) ^a^ |  |  |  |  |
| Living with a partner | 94 | 95 |  |  |
| Alone | 3 | 3 |  |  |
| Other | 4 | 2 | χ^2^ = 6.17 | **0.046** |
| Nulliparous, (%) | 46 | 39 | χ^2^ = 31.68 | **<0.001** |
| Fear of childbirth diagnosis, (%) | 13 | 9 | χ^2^ =38.78 | **<0.001** |
| Gestational diabetes, (%) | 26 | 25 | χ^2^ = 0.87 | 0.351 |
| *Notes:* Second cohort pregnancies are part of the excluded cohort. Test statistics: χ2 = Chi-square test; Z = Mann-Whitney U test. ^a^ Data available on 1714 women (59 %) in the excluded cohort. | | | | |

**Figure S1. A directed acyclic graph (DAG) of the relationships between factors relevant to the current study.**

**
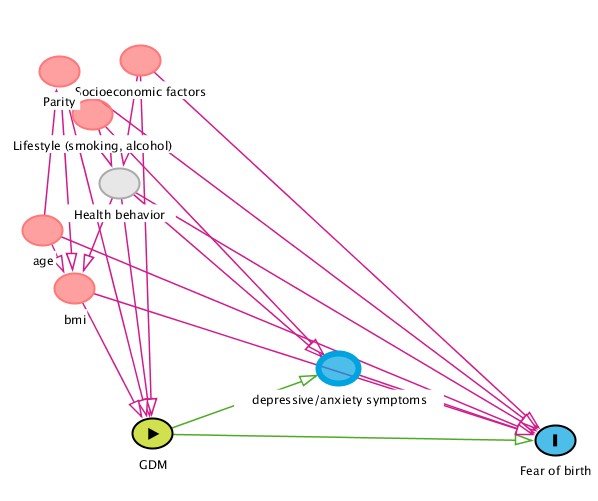
**
